# Perceived Elderly Stigma and Emotional Well-Being Among Korean Older Adults: The Mediating Role of Self-Esteem

**DOI:** 10.1093/geroni/igaa057.2168

**Published:** 2020-12-16

**Authors:** Eun Ha Namkung, Soondool Chung

**Affiliations:** 1 Brandeis University, Waltham, Massachusetts, United States; 2 Ewha Womans University, Seoul, Republic of Korea

## Abstract

This study examined whether self-esteem mediates the association between perceived elderly stigma and emotional well-being (loneliness and emotional isolation) among Korean older adults, and how these processes differ by marital status. Using the 2018 Age Integration Survey, a cross-sectional national survey of adults in Korea, we analyzed data from 266 older adults aged 60 and older. Older adults who perceived greater elderly stigma reported higher levels of loneliness and emotional isolation. Self-esteem played a significant indirect role in the association between perceived elderly stigma and the two emotional well-being outcomes. Moderated mediation analyses further revealed significant differences by marital status; self-esteem was a more powerful mechanism among unmarried older adults relative to their married counterparts. The findings suggest that efforts to minimize public and internalized stigmatization of older adults and to improve their own self-esteem may be critical for their emotional well-being.

